# On the improved electrochemistry of hybrid conducting-redox polymer electrodes

**DOI:** 10.1038/s41598-017-05063-z

**Published:** 2017-07-07

**Authors:** Louis Sieuw, Bruno Ernould, Jean-François Gohy, Alexandru Vlad

**Affiliations:** 1 Institute of Condensed Matter and Nanosciences, Bio- and Soft Matter, Université catholique de Louvain, Place Louis Pasteur 1, 1348 Louvain-la-Neuve, Belgium; 2 Institute of Condensed Matter and Nanosciences, Molecules, Solids and Reactivity, Université catholique de Louvain, Place Louis Pasteur 1, 1348 Louvain-la-Neuve, Belgium

## Abstract

The electrochemistry of poly(2,5-dihydroxyaniline) (PDHA), a novel hybrid molecular configuration with redox active sites and electrical charge conduction along the polymer chain, has been recently reported. The theoretical capacity of this material is estimated at 443 mAh g^−1^, with high power performances being proposed given the intrinsic electrical conductivity. However, the initial results were below the expectations: only half the theoretical capacity attained, poor cycling stability and modest power behavior calling for further investigations on improving these performances. Herein we detail the optimized chemical synthesis and electrode formulation for poly(2,5-dihydroxyaniline) resulting in improved cycling stability, power performances and defined electrochemical response. We also detail the alternative electrochemical synthesis and activation route for PDHA and compare the results with the chemical approach.

## Introduction

In the context of a sustainable energy future, Li-ion batteries are increasingly investigated^1–4^. Although inorganic battery materials have dominated this field for decades, organic redox compounds are gaining importance^5, 6^. These indeed present certain advantages over their inorganic counterparts. Organic compounds can be easily tailored in order to adjust their redox properties – notably, their redox potential can be adjusted with electron withdrawing and donating groups^6^ – and offer new possibilities for high energy and power density systems or hybrid devices^7, 8^. They are also constituted of abundant elements (C, H, O, N, S) and can be potentially generated from renewable resources, ensuring low cost and limited environmental impact^9^. The absence of transition metals in their composition ensures safer disposal, for example by incineration. Moreover, these materials are compatible not only with lithium chemistries, but also towards others, beyond-lithium technologies including sodium^10^, magnesium^11^ or zinc^12^.

Amongst organic redox active materials, quinone derivatives constitute a very promising class of Li-ion battery electrode materials, with electrochemical characteristics challenging those of conventional inorganic cathode materials^6, 13–15^. Various quinone-based formulations have been used so far with intrinsic advantages and drawbacks. The first and simplest quinone compound to be considered as electrode material is the 1,4-benzoquinone (BQ), with a theoretical capacity of about 500 mAh g^−1^ given the two-electron reversible redox and low molar mass^16^. However, the solubility of BQ in battery electrolytes is cause for major concerns. Different strategies have been proposed in the past to overcome this disadvantage, either by avoiding the use of a liquid electrolyte within a solid-state cell configuration^17^ or by limiting the solubility of the material. As part of the second option, surface grafting methods have been explored for BQ units^13, 18–20^, while others used BQ derivatives (for instance, dicarboxylates) with intrinsic limited solubility^9, 21, 22^. The drawback of the later is the increased molar mass leading to lower theoretical capacities. The third, and probably the most promising route to reduce the solubility is the use of polymeric quinones^23–27^.

**Figure 1:**
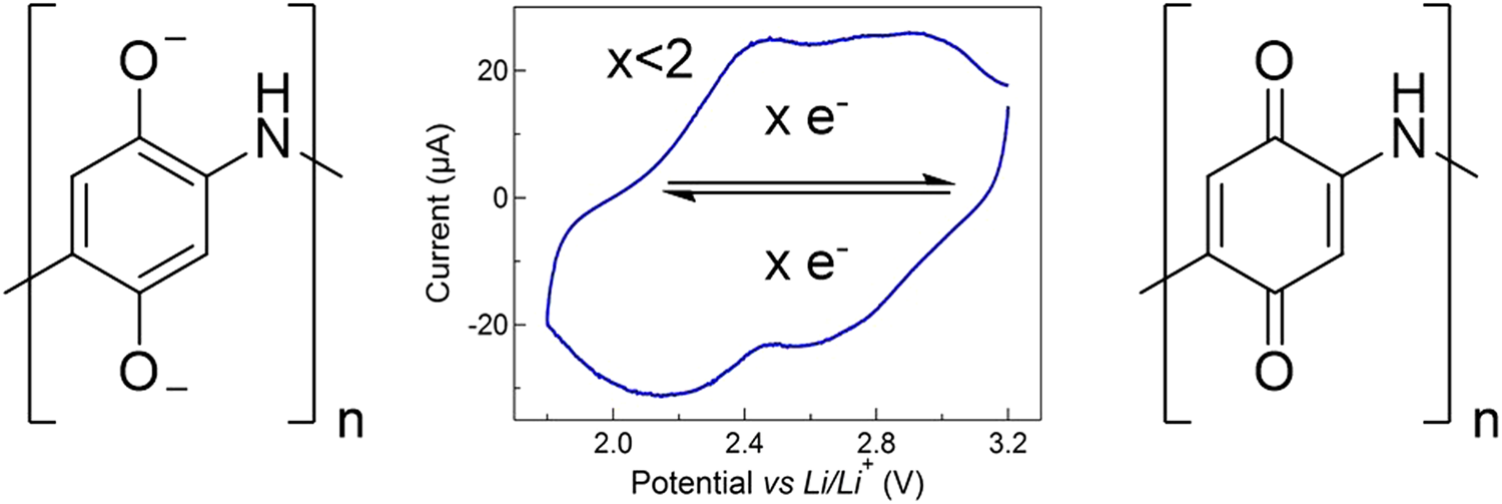
Molecular design and target electrochemistry of the hybrid redox node - electron-conducting PDHA.

It is this latter strategy that we explored in our recent work on poly(2,5-dihydroxyaniline) (PDHA)^15^. The molecular design of this BQ-based polymeric material (see Fig. 1) presents appealing properties for battery applications. The hybrid redox node - electron conducting chain structure was expected to enable simultaneous charge conduction and *n*-type redox while maintaining a low molar mass per repeating unit. Aside from its electrical conductivity, the polyaniline (PANi) backbone was also selected given the limited solubility of high molar mass polyanilines. The theoretical charge storage capacity for a two-electron redox process in BQ units of PDHA was estimated at 443 mAh g^−1^. Given the relatively high electrical conductivity of 100 mS cm^−1^ ^15^, PDHA was also advanced with potential for high power performances.

Despite the promising estimated energy metrics, the performances attained were below the expectations. The developed methodology did not allow us to reach the theoretical gravimetric capacity of the PDHA yet, a charge storage capacity of 270 mAh g^−1^ was still possible to attain. Other limitations were related to poor cycle life as well as to ambiguous electrochemical response. As for the capacity extraction, Poizot *et al*.^28^ postulated that structural aspects can induce the redox process to be limited to one-electron redox per BQ instead of the theoretical two. Furthermore, even though polyaniline is the oldest known synthetic conjugated polymer, its molecular structure is still matter of debate. The fact that polyaniline and its derivatives are displaying structures markedly influenced by the synthetic conditions is not helping to overcome this situation.

In this respect, the present work aims to get a better understanding of the limitations at play with PDHA in order to bring its performances to a higher level, particularly regarding the specific capacity and the capacity retention upon cycling. Therefore, the focus was set on several aspects that have not been explored in our first publication regarding the electrode formulation but also the material synthesis and processing. More specifically, we are now presenting parameters that have been reconsidered for the chemical demethylation step of poly-2,5-dimethoxyaniline (PDMA_CHEM_) in the chemical synthesis approach of PDHA_CHEM_. Additionally, we also explore and discuss the all-electrochemical synthesis route for PDHA_E-CHEM_^29–32^: electro-polymerization of the poly-2,5-dimethoxyaniline (PDMA_E-CHEM_), followed by electro-oxidative demethylation. Eventually, our attention has been focused on improving the active material-additives slurry formulation and electrode processing.

## Results and Discussion

### Synthesis

First, the results of the electrochemical synthesis of PDHA_E-CHEM_ are detailed. The motivation for this approach was that it can directly afford PDMA (and potentially PDHA) as an electrode, i.e. the pure deposited material without any additives (carbon, binder). Not only would this grant electrochemical cells with higher energy densities, but it also makes for a very simple and straightforward procedure. The electrodeposition conditions on stainless steel substrate (potential window, voltage sweep rate, number of scans) were first screened to obtain homogeneous, compact and adherent PDMA films.

**Figure 2:**
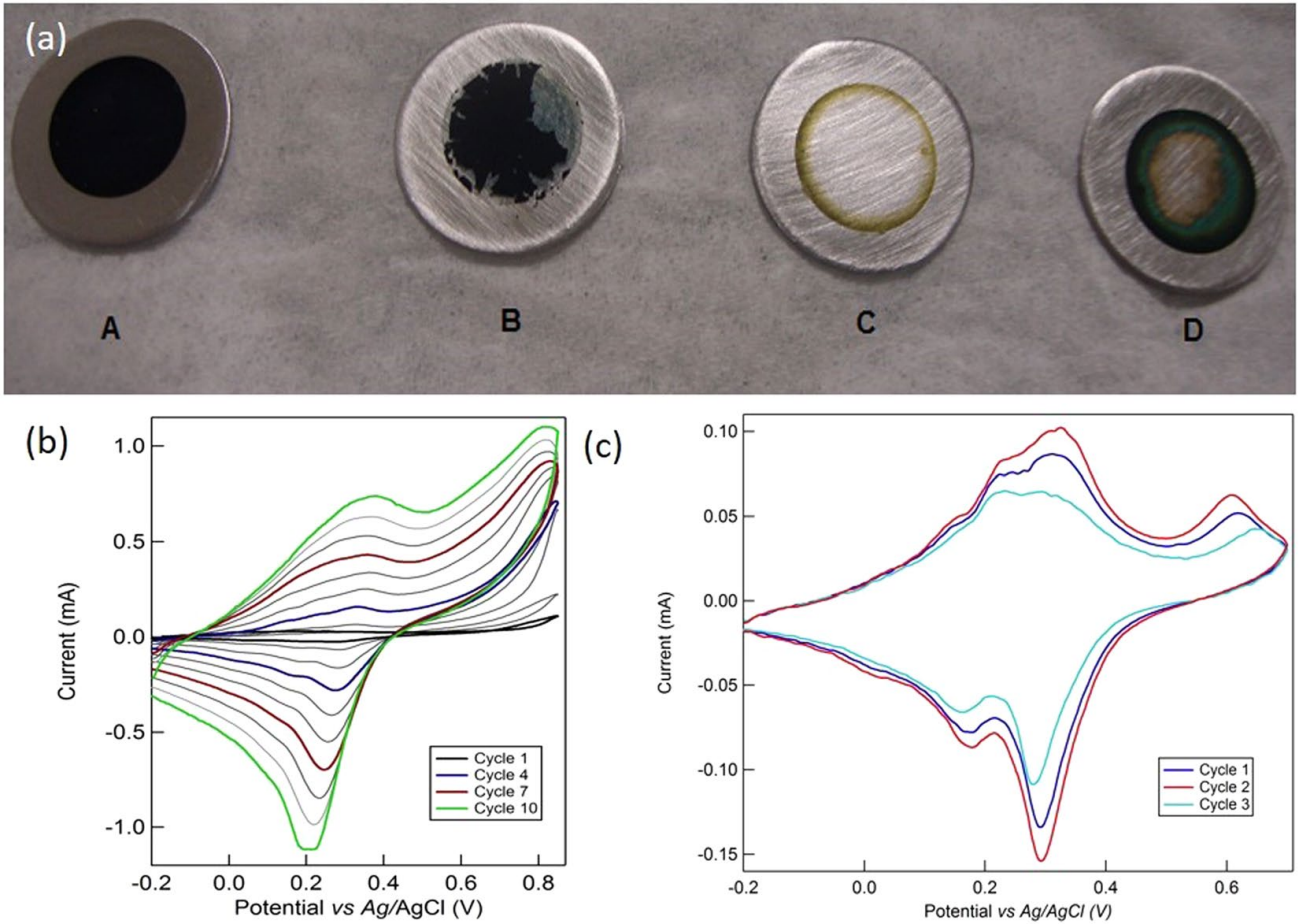
(**a**) Photographs of PDMA_E-CHEM_ films electrodeposited on stainless steel disks under various conditions (A**)** −0.2 to 0.85 V, 5 mV s^−1^, 10 scans; (B**)** −0.2 to 0.8 V, 5 mV s^−1^, 15 scans; (C) −0.2 to 0.8 V, 15 mV s^−1^, 5 scans; (D**)** −0.2 to 0.8 V, 10 mV s^−1^, 10 scans. Cyclic voltammogram for (**b**) typical PDMA_E-CHEM_ synthesis (scan rate 5 mV s^−1^) and (**c**) associated voltammogram for the electrochemical demethylation process (scan rate 1 mV s^−1^).

A series of typical samples and associated cyclic voltammograms for the electro-polymerization and the demethylation steps are shown in Fig. 2. The voltammogram in Fig. 2b shows progressive current increase with each cycle, significative of an increased amount of polymer being electrodeposited for each subsequent cycle. The porosity and thus the surface area increase allows for more monomer to be electro-oxidized, leading to a signal increase at every cycle. However, excessive deposition resulted in delamination of the PDMA film upon washing and drying, this step thus requiring further optimization. Films obtained after 10 scan cycles were found to be optimal (photographs A, B in Fig. 2a). A scan rate of 5 mV/s was also found optimal; for faster scan rates, non-uniform deposits were obtained (photographs B, C, D in Fig. 2a). Finally, the optimal scan potential window was determined to be −0.2 to 0.85 V *vs*. Ag/AgCl. The cyclic voltammogram of the electro-polymerization (Fig. 2b) displays a broad anodic peak around 0.8 V *vs*. Ag/AgCl, corresponding to the oxidation of the monomer and the electro-polymerization. The second anodic peak (0.3 V *vs*. Ag/AgCl) and the associated cathodic peak (0.2 V *vs*. Ag/AgCl), which are less visible at the beginning but become more pronounced with each cycle, are ascribed to the oxydo-reduction of the formed polymer. The most conclusive result (photograph A in Fig. 2a) in terms of homogeneity and structural integrity of the film was thus achieved with the following parameters: −0.2 to 0.85 V *vs*. Ag/AgCl, 5 mV s^−1^, 10 cycles. Subsequent analysis was carried out on PDMA_E-CHEM_ samples synthesized using these parameters.

After electro-polymerization, the samples were washed to remove any trace of monomer and electrochemical demethylation was tested. The conditions were similar to those of the polymerization except for the absence of monomer: voltammetry scans in 0.5 mol.L^−1^ H_2_SO_4_ aqueous electrolyte. The potential scan window was limited to −0.2–0.7 V *vs*. Ag/AgCl at a scan rate of 1 mV s^−1^ (Fig. 2c). Two sets of redox peaks can be distinguished on the voltammogram. The reversible waves at 0.2 and 0.3 V *vs*. Ag/AgCl are characteristic of the polymer backbone redox (leucoemeraldine - emeraldine couple). The peak at 0.65 V *vs*. Ag/AgCl is indicative only of an irreversible process that we assign to the oxidative demethylation of the PDMA. It should be noted that the emeraldine - (per)nigraniline redox potential in acidic solution was reported to be in the range of 0.65–0.85 V *vs*. Ag/AgCl with this process being partially reversible and leading to chain degradation when in presence of oxygen^33–35^. It is thus possible that the oxidative demethylation and the redox of the backbone are concurrent processes, leading to difficulties in precisely quantifying the specific contribution of each. Moreover, both processes can account for the irreversible chain degradation. It should also be noted that the peak at 0.65 V did not entirely vanish, even after extended scanning, which would have been expected in the case of complete demethylation of PDMA without interference of the emeraldine - (per)nigraniline redox process. Besides, the possibility of the chain degradation is supported by the global decrease of the whole signal after each voltammetry cycle. The physico-chemical and electrochemical characterizations that are following are providing some insight about the nature and the extent of the processes occurring during this synthetic approach.

Regarding our second approach, consisting in the chemical demethylation of PDMA_CHEM_, its yield was most comprehensively tuned by the amount of BBr_3_ equivalents as well as the reaction time. The demethylation yield was evaluated through elemental analysis (see the characterization section) and the capacity extracted from electrochemical measurements in coin cells (see the electrochemical characterization section). For one equivalent of methoxy groups, the yield increased significantly from 1.5 up to 3 equivalents of demethylating agent; going up from 3 equivalents, the yield gain was much less pronounced. Regardless of the excess of BBr_3_, the reaction is relatively slow, and a total time of 72 hours was finally retained to ensure a maximum demethylation yield.

### Characterization

**Figure 3:**
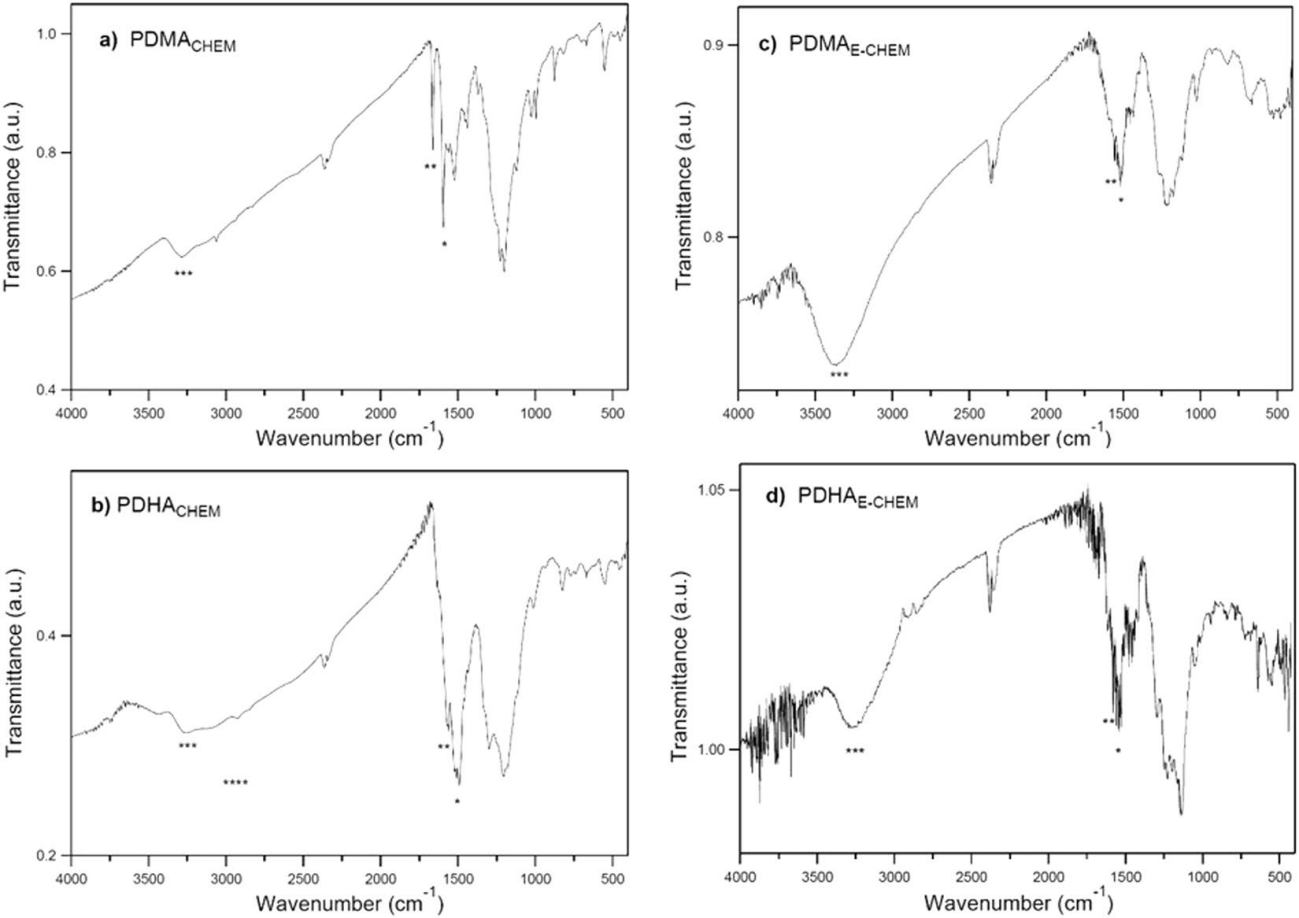
IR spectra of PDMA and PDHA synthesized by chemical and electrochemical approach: (**a**) PDMA_CHEM_, (**b**) PDHA_CHEM_, (**c**) PDMA_E-CHEM_ and (**d**) PDHA_E-CHEM_.

The electroactive polymers synthesized using both chemical and electrochemical methods were first characterized to determine the chemical composition and molecular structure. In both cases, the precursor polymer and the product of demethylation exhibited IR peaks around 1520 cm^−1^ (marked with * in Fig. 3) – stretching mode of the benzenoid ring – and 1590 cm^−1^ (marked with ** in Fig. 3) – stretching mode of the quinoid ring. The presence of the respective vibration modes supports the polymeric formula with polyaniline backbone in emeraldine oxidation state^36^. The N—H stretching peak (3300 cm^−1^) (marked with *** in Fig. 3) of the secondary amines of the polymer backbone is distinguishable for both PDMA_CHEM/E-CHEM_ and PDHA_CHEM/E-CHEM_. It is however more intense for PDMA_E-CHEM_ and PDHA_E-CHEM_, which hinders the observation of the O—H peak (marked with **** in Fig. 3) in the spectrum of PDHA_E-CHEM_ (Fig. 3d). This is not the case for the chemical synthesis route, and the O—H for PDHA_CHEM_ is clearly distinguishable (Fig. 3b), confirming the success of the demethylation reaction. This analysis suggests a higher demethylation yield for the chemical approach (PDHA_CHEM_).

To quantify the demethylation extent in PDMA_CHEM_/PDHA_CHEM_, elemental analysis was employed (Table 1) in addition to the capacities extracted from galvanostatic discharge (see the electrochemical characterization section). Outside the four main elements contained (C, H, N, O), the remaining percentage (17.51%) is attributed to Cl from the emeraldine salt. The obtained C/H, C/N and C/O ratios are compared to the theoretically estimated ratios based on molecular formulas for emeraldine form of PDMA (C_32_H_34_O_8_N_4_Cl_2_) and PDHA (C_24_H_18_O_8_N_4_Cl_2_). A clear trend after demethylation is the decrease of C/N and C/O ratios, accompanied by C/H ratio increase. The C/N, C/O and C/H ratios closest to the expected values were measured for PDHA_CHEM_ samples with the following demethylation conditions: 72 h at −5 °C with 3 or more equivalents of BBr_3_. However, the demethylation yield derived from this analysis shows rather important variations (55 to 130%) depending on the ratio on which we base our calculations (C/N, C/O or C/H). This lack of precision is most certainly due to the contamination from the substrate during the mechanical film peeling, as well as the limited amount of material available per single batch. The yields derived from the galvanostatic discharge capacities were in that regard more conclusive, as will be illustrated in the electrochemical characterization section.

**Table 1:** Elemental analysis. Atomic (C, H, N, O) percentages for PDMA_CHEM_ and PDHA_CHEM_.

	% C	% H	% N	% O	% rest	C/H	C/N	C/O
PDMA_THEOR_						0.94	8.00	4.00
PDMA_CHEM_	47.31	5.23	5.75	24.40	17.51	0.78	9.60	2.59
PDHA_THEOR_						1.33	6.00	3.00
PDHA_CHEM_	51.20	4.09	8.42	24.97	11.32	1.04	7.01	2.05

**Figure 4:**
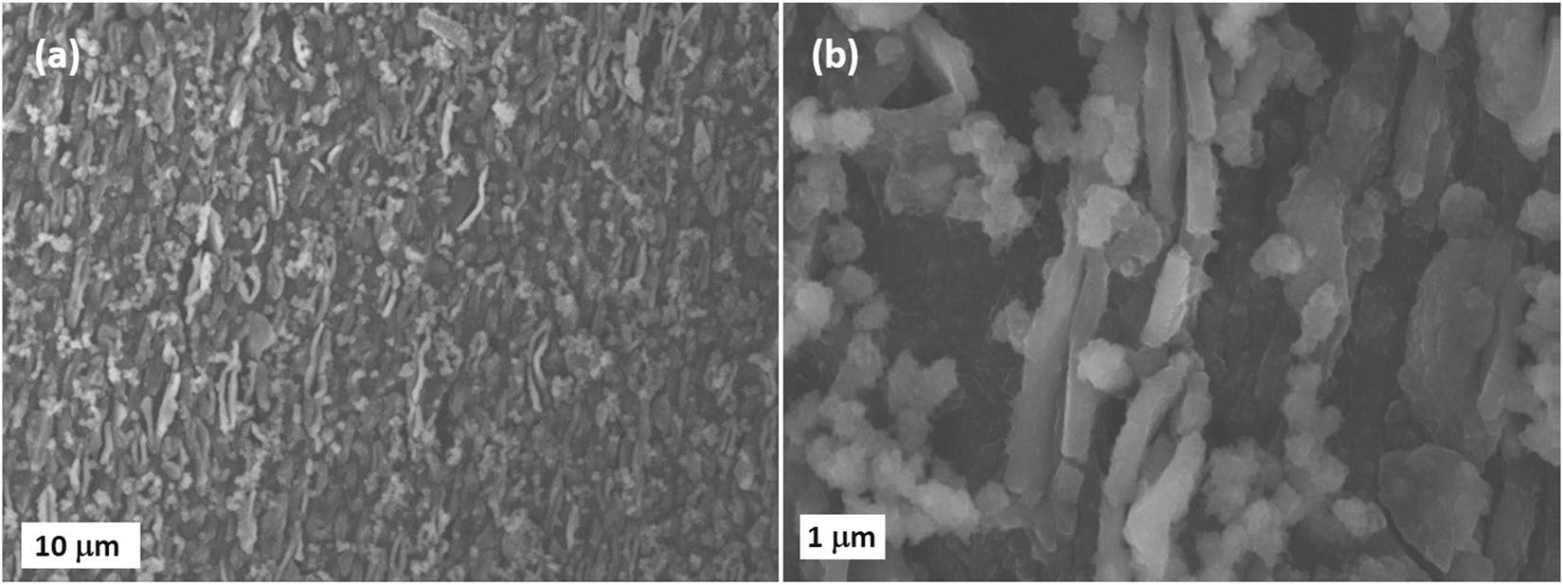
SEM images of PDHA_E-CHEM_ electrodes surfaces: 15 keV, SEI mode, WD = 8.4 mm (**a**) x1,000 (**b**) x6,000.

Finally, SEM analysis was carried out on PDHA_E-CHEM_ electrodes given that particle delamination was noticed during the electropolymerization (Fig. 4a,b). The polymer film is composed of nano- and micro-particles (1–10 μm) with indeed loosely bound particles present on the surface. The bulk film remains compact with no significative porosity being detected by SEM. The polymer film can however be easily swelled by a solvent, implying a mesoporous morphology. By allowing an easy access of the electrolyte to the active material, this should have a favorable impact on the electrochemical performances/properties that have to be studied next.

### Electrochemical characterization in non-aqueous electrolytes

**Figure 5:**
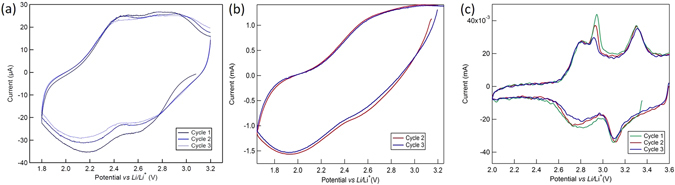
Cyclic voltammetry. (**a**) PDHA_CHEM_ - potential range: 1.8 to 3.2 V vs. Li/Li^+^, rate: 0.1 mV s^−1^. (**b**) PDHA_CHEM_ reported in ref. 15 - potential range: 1.8 to 3.2 V vs. Li/Li^+^, rate: 1 mV s^−1^. (**c**) PDHA_E-CHEM_ - potential range: 2.0 to 3.6 V vs. Li/Li^+^, rate: 1 mV s^−1^.

With both materials (PDHA_CHEM_ and PDHA_E-CHEM_) synthesized and characterized, we entailed electrochemical characterization in non-aqueous Li-half-cell configuration (Fig. 5a). Similarly to what was previously reported^15^, the half-cells typically had an open circuit potential of 2.8–3.2 V *vs*. Li/Li^+^ and required an initial cathodic polarization scan. The electrodes made of PDHA_CHEM_ display reversible behavior with two pairs of peaks centered at 2.3 and 2.7 V *vs*. Li/Li^+^, which are ascribed to the 2 electrons of the redox process, typical for quinone redox in aprotic electrolytes^6^. The absence of such peaks in pristine PDMA_CHEM_ electrodes^15^ constitutes an indication of successful demethylation. For comparison, the CV of the previously reported PDHA_CHEM_ material^15^ is shown in Fig. 5b. A visible improvement concerning the definition of the redox response is observable, implying a higher demethylation yield obtained using the current method than with the previous reaction parameters. This also corroborates the higher capacities obtained on galvanostatic cycling of PDHA_CHEM_ synthesized in this work, involving higher activation of quinone redox moieties.

In contrast, the electrochemical analysis of PDHA_E-CHEM_ shows a different behavior from PDHA_CHEM_. Two redox waves– centered at 2.8 and 3.2 V *vs*. Li/Li^+^ – can be distinguished (Fig. 5c). The processes are reversible, with a slight decrease in intensity of the peak at 2.8 V observed with increasing cycle number. It should be noted that others have also attempted the electrochemical synthesis of PDHA for charge storage applications^29–32^. However, we find a lower redox activity window for PDHA_E-CHEM_: 2.5 to 3.6 V *vs*. Li/Li^+^ compared to the 3.0–4.0 V *vs*. Li/Li^+^ window previously reported. The assigned redox peak values are also not consistent with the electrochemistry of benzoquinone in non-aqueous electrolytes. The redox process centered at 3.2 V *vs*. Li/Li^+^ is characteristic of the redox reaction of the electroactive polyaniline chain (refer also to Fig. 2b,c)^37, 38^. While the peak centered at 2.8 V *vs*. Li/Li^+^ could be assigned to PDHA redox, the absence of the second associated redox peak (typically located between 2.0 and 2.6 V *vs*. Li/Li^+^ as seen in Fig. 5a,b) could be an indication that we are confronted with a different redox process than the classical quinone redox couple.

The explanation for this could be the high anodic polarization required for PDMA demethylation that also increases the redox state within the polyaniline chain. When in presence of oxygen, the (per)nigraniline has been reported unstable with irreversible degradation of the polymer matrix. The additional presence of methoxy/hydroxyl groups here could lead to complex electrochemical stability - response interplay requiring further investigation. Overall, this analysis suggests that the electrochemical synthesis route of PDHA requires further optimization to reach results similar to the chemical demethylation approach.

Lastly, galvanostatic cycling tests were run on PDHA_CHEM_ assembled electrodes to determine the attainable reversible capacities and cycling stability. PDHA_CHEM_ half-cells were cycled within a potential range from 1.8 to 3.0 V *vs*. Li/Li^+^. The voltage window was preliminarily determined by CV (Fig. 5a) and the upper limit was set to 3 V to avoid any overlap with the polyaniline backbone redox. The specific capacities, directly linked to the demethylation yields, varied with the demethylation conditions, as illustrated in Table 2. Higher excesses of BBr_3_ demethylating agent and longer reaction times allow for an improved conversion of methoxy groups, and thus a higher amount of hydroxy chemical functions accessible for the electrochemical process. Accordingly, we observe an uptake of the specific capacity when these two reaction parameters are increased.

**Table 2:** Demethylation conditions for the PDMA_CHEM_ and corresponding specific capacities for PDHA_CHEM_.

PDHA_CHEM_ batch	BBr_3_ (eq)	t (h)	Specific capacity (mAh g^−1^)
1	1,5	24	120
2	1,5	72	165
3	3,5	72	290

**Figure 6:**
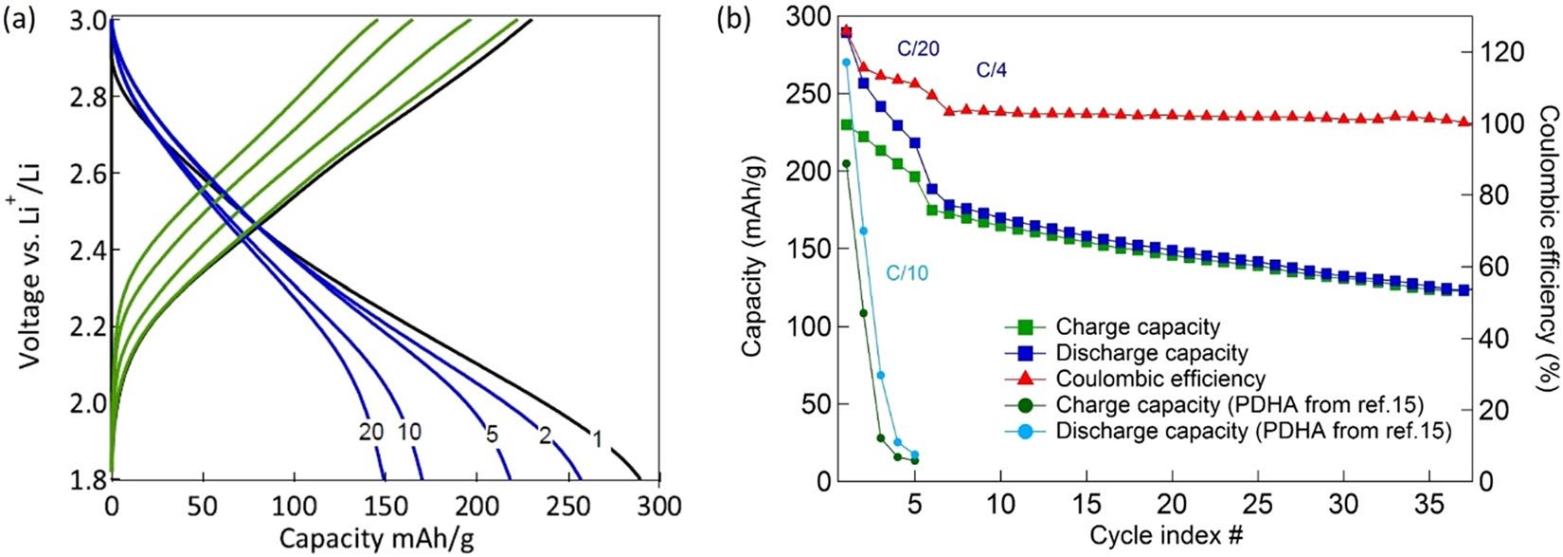
Galvanostatic charge-discharge tests of PDHA_CHEM_ electrodes. (**a**) Voltage vs. capacity profiles for PDHA_CHEM_ electrodes. (**b**) Capacity retention plot for PDHA_CHEM_ electrodes.

The best demethylation conditions led to an initial discharge capacity of 290 mAh g^−1^ (Fig. 6a). This constitutes the highest to date capacity for this class of materials, and is attributed to improved synthesis, limited exposure to air of the demethylated material and storage in controlled atmosphere prior utilization.

Another major improvement is the enhancement of the capacity retention (Fig. 6a,b). For instance, 90% of the initial capacity (270 mAh g^−1^) was lost in the first three cycles for the highest capacity PDHA previously reported^15^. As illustrated in Fig. 6b, the PDHA_CHEM_ electrodes analyzed here retain more than 50% of the initial capacity after more than 30 cycles. These ameliorations are the result of an improved synthesis method, an adequate current collector implementation (carbon-coated aluminum foil here *vs*. nickel foam in ref. 15), and an improved adhesion of the coated electrodes.

The coulombic efficiency for the PDHA_CHEM_ electrodes is also illustrated in Fig. 6b, with initial values above 100%. High reversibilities of carbonyl groups have been reported before for other organic electrode materials, with associated good coulombic efficiencies^9, 24, 25, 39, 40^. In this case, the excessive values obtained for a current density of 16 mA g^−1^ (C/20) are ascribed to irreversible redox processes in the polymer backbone. With cycling, the coulombic efficiency values stabilizes at 100%, which implies limited irreversible redox of the backbone.

**Figure 7:**
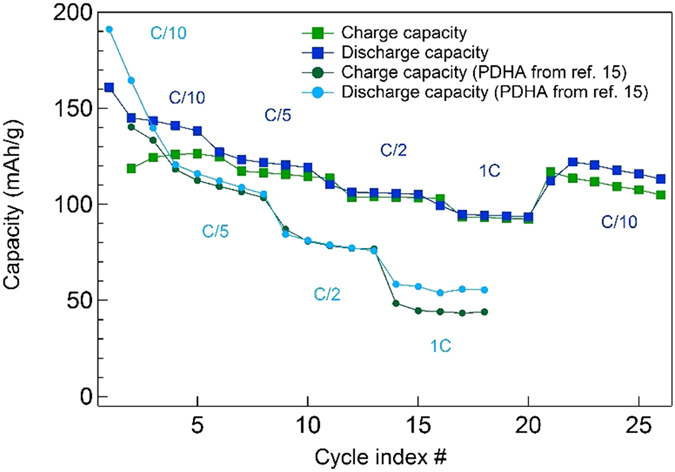
Variable rate capacity retention plot for PDHA_CHEM_ and PDHA reported in ref. 15.

Lastly, the power performances of PDHA_CHEM_ have also been measured. Figure 7 displays the stable reversible capacity obtained - the electrodes delivered specific capacities of 160, 130, 110 and 100 mAh g^−1^ at 16 (C/10), 32 (C/5), 80 (C/2) and 160 mA g^−1^ (1C), respectively. Moreover, after the power-rate evaluation, the PDHA_CHEM_ retained most of the initial capacity of 125 mAh g^−1^, thus 80% of the initial value. For comparison, the best rate performances achieved in our previous report are also shown. Despite a lower initial value at C/10 (160 mAh g^−1^ *vs*. 190 mAh g^−1^), the capacities delivered at C/2 (110 mAh g^−1^ vs 85 mAh g^−1^) and 1C (100 mAh g^−1^ vs 60 mAh g^−1^) are significantly higher in current work. These improved power properties are ascribed to optimized synthesis and formulation parameters, and notably to the change of current collector with carbon-coated aluminum foil used this time instead of Ni foam.

## Conclusion

To summarize, this work details the chemical and electrochemical synthesis of poly(2,5-dihydroxyaniline) coupled to electrochemical analysis of the synthesized materials. Full and reversible capacity utilization in this hybrid redox-conductive polymer still remains elusive with however some important insights being presented in this work. The electrochemical method was tested as it can provide pure PDMA (and potentially PDHA) electrodes to truly benefit from intrinsic electrical conductivity, electrolyte insolubility and high capacity of this material. Nevertheless, the electroanalytical analysis of PDHA_E-CHEM_ points towards a mechanism differing from the demethylation, with possible enhanced degradation given the high anodic polarization required. Further improvement of the electrochemical demethylation method is thus required for it to deliver more satisfactory results. The optimization of the chemical synthesis, electrode processing and assembly protocol in turn has led to clear improvements in terms of first cycle capacity, cycle capacity retention and power performance. This work is expected to lead to further progress in this direction by enabling full capacity utilization in PDHA and gives guidelines for new alike hybrid redox-conductive polymers materials for energy storage.

## Methods

### Materials and reagents

The 2,5-dimethoxyaniline monomer (C_8_H_11_O_2_N, DMA, Acros Organics, 99+%) was sublimed twice at 80 °C under reduced pressure prior to polymerization. Ammonium Persulfate ((NH_4_)_2_S_2_O_8_, Acros Organics, 98%), hydrochloric acid (HCl, Fisher chemical, 37%), diethyl ether ((C_2_H_5_)_2_O, VWR, GPR), ammonium hydroxide (NH_4_OH, ChemLab, 28–30%), boron tribromide (BBr_3_, Sigma Aldrich, 99.9%) and sulfuric acid (H_2_SO_4_, Sigma Aldrich, 98%) were used as purchased. Dichloromethane (CH_2_Cl_2_, VWR, HPLC grade) was deoxygenated by argon purging before utilization.

### Electrochemical synthesis approach

The electrochemical synthesis was carried out in a one-compartment three-electrode cell. A polished stainless steel disk (SS-304) was used as the working electrode, a platinum wire as the counter electrode and an Ag/AgCl electrode as reference electrode. All the experiments were performed using an Arbin instruments BT – 2043 multichannel potentiostat. PDMA_E-CHEM_ films were deposited by means of cyclic voltammetry from an aqueous solution containing 0.05 mol L^−1^ DMA and 0.5 mol L^−1^ H_2_SO_4_. The potential range, the scan rate and the number of scans were optimized in order to obtain adherent and homogeneous deposits. After the electro-polymerization, the films were rinsed with water and dried.

**Figure 8:**
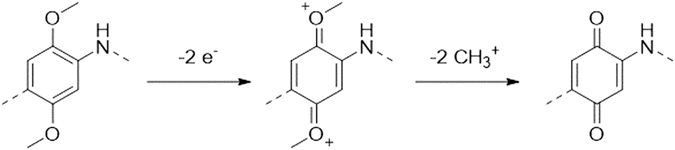
Electrochemical approach for the demethylation of PDMA_E-CHEM_ into PDHA_E-CHEM_.

For the oxidative demethylation (yielding PDHA_E-CHEM_), the obtained PDMA_E-CHEM_ films on stainless steel substrates were used as the working electrode and exposed to cyclic voltammetry polarization scans in a 0.5 mol L^−1^ H_2_SO_4_ aqueous solution. After several optimization tests, the following parameters were found as optimal: the potential was cycled between −0.2 and 0.7 V *vs*. Ag/AgCl with a scanning rate of 1 mV s^−1^. The mechanism for the electrochemical demethylation reaction (Fig. 8) proceeds through the oxidation of PDMA_E-CHEM_ (schematized here for the 2 electrons reaction per monomer unit), followed by the elimination of two methyl cations.

Chemical demethylation of PDMA_E-CHEM_ was also tested but unsatisfactory results have been obtained. During the reaction in conditions similar to above, the stainless-steel disk displayed signs of corrosion (progressive appearance of a dark-brown film/deposit) accompanied by delamination of the PDMA film.

### Chemical synthesis approach

**Figure 9:**
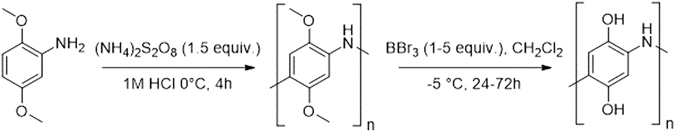
Chemical approach for the synthesis of PDHA.

The previously reported chemical synthesis of PDHA was reconsidered here^15^. The method consists in (i) oxidative polymerization of 2,5-dimethoxyaniline using ammonium persulfate as oxidizing agent to obtain PDMA, followed by (ii) chemical demethylation of PDMA with boron tribromide to give PDHA^15^ (Fig. 9).

The PDMA_CHEM_ was obtained following the literature reports^33, 41, 42^. In short, DMA (0.03 mol L^−1^) was dissolved in 1 mol L^−1^ HCl aqueous solution followed by slow addition of ammonium persulfate ((NH_4_)_2_S_2_O_8_, 1.5 equivalents) as oxidizing agent at 0 °C. The reaction was further stirred for 4 h at 0 °C. The obtained PDMA_CHEM_ was retrieved by centrifugation and washed with 0.1 mol L^−1^ HCl and diethyl ether. The polymer (yield – 75%) was further stored under argon atmosphere.

For the chemical hydrolysis of the methoxy groups, the reaction time, temperature and the amount of demethylating agent were screened to find the optimal conditions. First, PDMA_CHEM_ was basified with an ammonium hydroxide solution (NH_4_OH 0.1 mol L^−1^) then dried overnight under reduced pressure at 35 °C. The PDMA_CHEM_ was then dispersed in dichloromethane (with also partial dissolution) and the solution was cooled to −5 °C. Afterwards, boron tribromide (BBr_3_) (1.5 to 5 equivalents) was added dropwise over 1 hour. The reaction time varied from 24 to 72 hours while maintaining the temperature at −5 °C. The reaction was quenched with deionized water, recovered by vacuum filtration and washed with 0.1 mol L^−1^ HCl and diethyl ether. The obtained product (PDHA_CHEM_) displayed the expected green color (acidified form of polyanilines) and solid consistency, whereas the solubility in water and acetonitrile considerably decreased compared to the PDMA_CHEM_ precursor.

### Physico-chemical characterization

FTIR spectra of PDMA/PDHA_CHEM/E-CHEM_ products were acquired with a Bruker Equinox 55 spectrometer by mixing the powders with KBr. Elemental analysis was performed by soliciting Medac Ltd services, with a stated absolute precision of 0.3%. Scanning Electron Microscopy (SEM) images were acquired using a LEO 982 FEG-SEM.

### Electrode formulation and electrochemical testing

To prepare the electrodes, PDHA_CHEM_ was first ball-milled with carbon black (SC45, Timcall, MTI). It should be mentioned that similarly to our previous work, carbon black was primarily used as reinforcement filler material to enhance the structural integrity of the coatings. Without added carbon black, coatings were found to crack and delaminate from current collectors even in presence of excess amount of binder. Two binder formulations have been tested: PVDF-NMP (poly(vinylidene fluoride) dissolved in N-methylpyrrolidone) and CMC/SBR-water (aqueous solution of carboxymethyl cellulose and styrene-butadiene rubber). The best results in terms of coating integrity during manipulation as well as post-cycling analysis were obtained using the formulation containing PDHA_CHEM_:SC45:PVDF − 30:60:10 (mass ratio) coated on carbon-coated aluminum foil (EQ-CC-Al-18u-260, MTI) as current collector. Attempts were made to reduce the proportion of carbon, but resulted in an important loss of integrity of the electrode during handling, cycling, and first cycle irreversible capacity loss.

Pure PDHA electrodes were prepared by means of electrochemical synthesis as detailed in the synthesis section. The PDHA_E-CHEM_ on SS disks were used as such without any further processing or additive incorporation. PDHA_CHEM/E-CHEM_ electrodes were tested in half-cell configuration using lithium metal (Gelon) as reference and counter electrode. A porous polyethylene membrane (Celgard) was used as separator. A mixture of ethylene carbonate (EC), diethyl carbonate (DEC) and dimethylcarbonate (DMC) with a 1:1:1 volume ratio with 1 mol L^−1^ of lithium hexafluorophosphate (LiPF_6_) was used as electrolyte (Solvionic). Electrochemical cells were assembled in nutlock-type cells (X2 Labwares). The assembly was made under protective argon atmosphere (<0.1 ppm H_2_O, <0.1 ppm O_2_) in a MBraun glovebox. All the galvanostic cycling experiments were performed using an Arbin BT – 2043 multichannel potentiostat.
